# Prednisolone at a therapeutic dose is not detrimental to mouse oocyte competence

**DOI:** 10.5935/1518-0557.20240108

**Published:** 2025

**Authors:** Shruthivishali Muthukumar, Vijeta Shetty, Akshatha Daddangadi, Satish Kumar Adiga, Shubhashree Uppangala

**Affiliations:** 1 Centre of Excellence in Clinical Embryology, Department of Reproductive Science, Kasturba Medical College, Manipal, Manipal Academy of Higher Education, Manipal-576 104, India; 2 Division of Reproductive Genetics, Department of Reproductive Science, Kasturba Medical College, Manipal. Manipal Academy of Higher Education, Manipal-576 104, India

**Keywords:** assisted reproductive technology, glucocorticoids, oocytes, prednisolone, preimplantation embryo development

## Abstract

**Objective:**

To examine the impact of PRDL on mouse oocyte developmental competence.

**Methods:**

This study was conducted on 6-8-week-old female Swiss albino mice in which different doses of PRDL were administered for three days during superovulation. Oocytes were assessed for quality, and subsequently, embryos derived from these oocytes were evaluated at the blastocyst stage. Simultaneously, oocytes were also exposed to PRDL during in vitro maturation and examined for their spindle and activation potential.

**Results:**

Compared to control group, the total cell number of blastocysts in the PRDL group increased. Nevertheless, the incidence of apoptosis was comparable between the groups. However, direct exposure at 10 µM significantly increased spindle abnormalities compared to those in the control group (*p*<0.05).

**Conclusions:**

Short-term PRDL exposure at a therapeutic dose is not detrimental to oocyte developmental potential in vitro. Although the current study confirmed the safety of PRDL in a mouse model, further studies involving long-term exposure are warranted, as patients receive PRDL treatment for extended periods of time.

## INTRODUCTION

The off-label use of glucocorticoids (GCs) as an adjuvant therapy during ovarian stimulation has attracted increased interest in recent years (Beltrán *et al.*, 2020; Conforti *et al.*, 2021; Farzaneh & Afshar, 2020). During the peri-implantation phase, both natural and synthetic GCs are believed to improve the uterine environment as immunomodulators (Boomsma *et al.*, 2022; Robertson *et al.*, 2016), whereas during the follicular phase, GCs ameliorate follicular development by suppressing androgen levels and increasing the production of growth factors, which are known to amplify the action of gonadotropins (Langford & Miell, 1993; Miell *et al.*, 1993). Low doses of GCs (10 mg/day) have been shown to have a positive impact on pregnancy, while women experiencing recurrent miscarriages and other conditions of immunological infertility have also been treated with higher doses of GCs (up to 60 mg/day) (Boomsma *et al.*, 2007; Kalampokas *et al.*, 2017). Nevertheless, there is scarce evidence regarding the efficacy of administering GCs during ovarian stimulation to increase live birth rates in women undergoing assisted reproductive technology (ART) treatment (Kalampokas *et al.*, 2017; Kim, 2021; Lin *et al.*, 2023).

Animal studies on the effect of GC exposure on oocyte maturation and developmental competence have shown conflicting results. Low-dose corticosterone administration in mice caused no adverse effects on oocyte maturation; however, the administration of higher concentrations of corticosterone caused alterations in oocyte ultrastructure and increased mitochondrial mutations (Li *et al.*, 2018). Cortisol administration impaired mouse oocyte developmental competence by inducing apoptosis (Yuan *et al.*, 2016; 2020). In contrast, supplementation of culture media supplemented with dexamethasone and cortisol improved oocyte maturation and development competence in bovine oocytes (Barroso *et al.*, 2020; da Costa *et al.*, 2016), but a negative effect of both cortisol and dexamethasone was observed on lamb and pig oocyte maturation (González *et al.*, 2010a; Yang *et al.*, 1999). In addition, high concentrations of corticosterone significantly affect oocyte maturation, fertilization and embryo development in vitro in a mouse model (Andersen, 2003; Čikoš *et al.*, 2019; González *et al.*, 2010b).

Prednisolone (PRDL) is a synthetic glucocorticoid given to patients with infertility conditions to ameliorate the ovarian response and pregnancy outcome in ART treatment (Revelli *et al.*, 2008); it is either given alone or in combination with other drugs (Duvan *et al.*, 2006; Ubaldi *et al.*, 2002). PRDL is widely prescribed for women with recurrent miscarriages or immunological infertility conditions to improve implantation outcomes (Robertson *et al.*, 2016). PRDL treatment may start a month before controlled ovarian stimulation (COS) (Geva *et al.*, 2000), during COS (Duvan *et al.*, 2006) or just after oocyte retrieval (Motteram *et al.*, 2015). PRDL is additionally employed during the COS protocol for patients with polycystic ovarian syndrome (PCOS) (Fridström *et al.*, 1999; Mohammadi Yeganeh *et al.*, 2018) and ovarian hyperstimulation syndrome (OHSS) (Fang *et al.*, 2021; Weigert *et al.*, 2002). Importantly, when used during the peri-implantation phase, PRDL treatment has specific adverse effects on preimplantation embryos, fetuses, and placentas in mice (Kieffer *et al.*, 2020; Uppangala *et al.*, 2021). However, no experimental studies have explored the effect of PRDL on oocyte maturation and developmental competence.

Recent meta-analyses have suggested that there is no added value in the use of PRDL in IVF cycles (Lin *et al.*, 2023). The clinical benefits of PRDL are debatable, and further studies are warranted to understand the impact of PRDL on oocytes exposed during the follicular phase. The main research question is whether there is any negative impact of PRDL on oocyte functional and genetic competence influencing its developmental competence and genetic integrity. Therefore, to bridge the knowledge gap on the impact of PRDL on oocyte maturation and developmental competence, we investigated the effect of PRDL administration during ovarian stimulation on oocyte maturation and the genetic integrity of embryos derived from oocytes exposed to PRDL. In our study, mice were administered different concentrations of PRDL (0.01, 0.1 and 1mg/kg body weight) for three days during the superovulation regimen to mimic the clinical scenario, and the oocyte number, maturation, mitochondrial potential, developmental competence and genetic integrity of the blastocysts were assessed. Additionally, to understand the direct effects of PRDL on oocyte maturation, immature mouse oocytes were exposed to PRDL at various concentrations (0.1, 1 and 10µM) for 24 h during in vitro maturation.

## MATERIAL AND METHODS

### Animals and ethics declaration

Healthy Swiss albino mice aged 6-8 weeks (female, n=85; male, n=25) maintained at 25±2°C and 45-55% humidity with a 12:12 h photoperiod and fed a standard diet and water ad libitum were used for the experiments. Animal handling and procedures were carried out as per the institutional guidelines, and the study was approved by the institutional animal ethics committee (IAEC/KMC/14/2021 and IAEC/KMC/16/2021).

### Preparation of prednisolone (PRDL)

A stock solution of 3 mM prednisolone (PRDL, Cat No. P6004, Sigma Aldrich, USA) was prepared using absolute ethanol (Cat No. F204325, Hayman, UK) as previously described (Uppangala *et al.*, 2021). For the in vivo approach, working concentrations of PRDL (0.01, 0.1 and 1 mg/kg body weight) were prepared by diluting the stock solution with phosphate-buffered saline (PBS). According to Kieffer *et al*. (2020), 1 mg/kg of PRDL in mice corresponds to the highest dose used in ART (60 mg/day). Hence, we used concentrations of 0.01, 0.1 and 1 mg/kg, where 0.01 mg/kg corresponds to the physiological concentration of GCs and 0.1 corresponds to the therapeutic dose (5-10 mg/day). For the in vitro approach, the stock solution was further diluted to 0.1, 1 and 10 µM PRDL using in vitro maturation (IVM) medium. A concentration of 0.1 µM corresponds to the physiological level of GCs (Burkuš *et al.*, 2013; Gong *et al.*, 2015), 1 µM is close to the therapeutic dose (5-6 mg/day), and 10 µM is ten times the therapeutic dose and is hence considered the supraphysiological concentration.

### PRDL administration and Cumulus oocyte complex (COC) collection

To mimic the clinical scenario, PRDL administration (intraperitoneal) started on the day of pregnant mare serum gonadotropin (PMSG) injection. Furthermore, PRDL was given for 3 days, with a 24 h time interval between each administration during the superovulation regimen. For superovulation, 5 IU PMSG (Cat No. HOR-272, ProSpecTany TechnoGene Ltd., Israel) followed by 10 IU human chorionic gonadotropin (hCG) (Cat No. Lupi-HCG 2000, Lupin, India) was administered to female mice at 48 h intervals. An approximately 8 h time interval was maintained between PRDL and PMSG injection as well as between PRDL and hCG administration. The animals were sacrificed 12 h after hCG injection, and COC was collected. COCs were either denuded to assess oocyte number, nuclear maturity, fragmentation and mitochondrial potential or subjected to IVF to assess oocyte developmental competence.

### Oocyte denudation and morphological assessment

The COCs were enzymatically denuded by treatment with 1 mg/mL hyaluronidase (Cat No. H4272, Sigma Aldrich, USA) for 30 seconds, and the denuded oocytes were assessed morphologically under an inverted phase contrast microscope (IX73, Olympus, Japan) to determine nuclear maturity and morphological abnormalities.

### Measurement of oocyte mitochondrial potential by JC-1 staining

To determine whether PRDL alters oocyte mitochondrial potential, PRDL-exposed oocytes were subjected to JC1 staining as described previously (Uppangala *et al.*, 2015). Briefly, oocytes were incubated with prewarmed 1µg/mL 5,5′,6,6′-tetrachloro-1,1′,3,3′-tetraethyl-imidacarbocyanine iodide (JC-1, Cat No. T3168, Molecular Probes, Life Technologies, USA) for 30 min at 37°C and 5% CO_2_. After 30 min, the excess probe was removed by washing with M16 medium supplemented with 0.1% BSA, and the plates were mounted on glass slides. The oocytes were observed under a fluorescence microscope (Imager-A1; Zeiss, Gottingen, Germany). Oocytes with low mitochondrial potential fluoresce green, while oocytes with high mitochondrial potential fluoresce orange owing to the aggregation of JC-1. The mitochondrial membrane potential is represented as the ratio of orange to green fluorescence, which was determined by ImageJ software (National Institute of Health, Bethesda, Maryland, USA).

### In vitro fertilization (IVF) and embryo culture

The COCs retrieved from the oviduct were incubated in potassium simplex optimization medium (KSOM) supplemented with 0.1% BSA (Cat No. A3311, Sigma Aldrich, USA) at 37°C and 5% CO_2_. Caudal sperm retrieved from male mice were subjected to swim up for 45 min at 37°C and 5% CO_2_ in KSOM supplemented with 1% BSA. The motile sperm fraction postswim-up was coincubated with COC. Oocytes were denuded and assessed for fertilization 10 h post insemination (hereafter referred to as hpi). Normally fertilized oocytes were cultured at 37°C and 5% CO_2_ in 20 µL of KSOM supplemented with 0.1% BSA and overlaid with paraffin liquid light oil. Preimplantation embryo development was assessed at regular intervals until 96 hpi. The total cell number and DNA integrity of the blastocysts were assessed.

### DNA damage assessment by terminal deoxynucleotidyl transferase (TdT) dUTP nick end labeling (TUNEL) assay

The TUNEL assay was performed as described previously (D’Souza *et al.*, 2016) with slight modifications. Briefly, at 96 h, blastocysts were fixed using 4% PFA overnight at 4°C in 60-well plates (Cat No. 163118, Nunc, India). The fixed blastocysts were permeabilized for 1 h using 0.1% Triton-100, 0.1% sodium citrate and 0.5% BSA in PBS. Later, the embryos were incubated for 1 h with a TUNEL reaction mixture (Cat No. 12156792910, Roche Diagnostics, Germany) in the dark at 37°C. After washing, the slides were counterstained with 4µg/mL DAPI and mounted onto a clean slide using mounting medium (Cat No. S3023, DAKO, Denmark). The embryos were observed under a fluorescence microscope (x400 magnification; Imager-A1; Zeiss, Gottingen, Germany) and imaged using Q-Capture software (Media Cybernetics Inc., USA). TUNEL-labeled cells displayed red fluorescence, whereas the nuclei of the cells displayed blue fluorescence. The TUNEL index was calculated by calculating the percentage of TUNEL-labeled cells relative to the total number of cells present in each embryo.

### Germinal vesicle (GV) oocyte isolation and PRDL treatment in vitro

To study the direct effects of PRDL on mouse oocytes, GV oocytes were collected from ovaries excised from female mice in prewarmed homemade M2 media. The cumulus-free GV oocytes isolated from the five study groups were randomly divided and cultured in vitro for 24 h in IVM medium supplemented with (0.1, 1 or 10 µM) or without PRDL.

Briefly, in vitro maturation of GV oocytes was carried out in 20 µL droplets of IVM medium with oil overlay for 24 h under growth conditions (37°C and 5% CO_2_). IVM medium was prepared by adding 0.05% pyruvate (Cat No. P3662, Sigma Aldrich, USA), 1% nonessential α-amino acids (Cat No. 11140-050, Gibco, USA), 1% ITS (Cat No. 51500-056, Gibco, USA), 0.05% penicillin‒streptomycin (Cat No. 15140-122, Gibco, USA) and 0.3% bovine serum albumin (BSA) to Dulbecco’s modified Eagle’s medium (DMEM). After IVM, oocytes were assessed for the presence of an extruded polar body, which indicates the maturity of the oocyte.

### Spindle integrity assessment

Mature oocytes obtained post IVM were examined for spindle morphology as per an earlier protocol (Daddangadi *et al.*, 2020). Briefly, oocytes were incubated with extraction buffer containing 50mM potassium chloride, 5 mM ethylenediaminetetraacetic acid disodium salt, 0.5mM magnesium chloride, 25% glycerol, 25mM HEPES, 20 µM phenyl methane sulfonyl fluoride and 2% Triton X-100, adjusted to pH 6.75 for 1 h under growth conditions. Oocyte fixation was performed at -20°C by exposing the oocytes to ice-cold ethanol for 12 minutes. Furthermore, the cells were treated with 0.25% Triton-X and 5% knockout serum (Cat No. 10828-010, Gibco, India) for 1 h at 37°C. Furthermore, the oocytes were incubated with a primary anti-α-tubulin antibody (1:150) (Cat. No. T9026, Sigma Aldrich, USA) overnight at 4°C. After washing, the oocytes were incubated with a secondary goat anti-mouse IgG antibody (1:500) (Cat No. NB7535, Novus Biologicals, USA) for 1 h at 37°C. After counterstaining with 4 µg/mL DAPI (40,6-diamidino-2-phenylindole, Cat. no. D9542, Sigma Aldrich, USA), the spindle was observed under a 40X objective of fluorescence microscope (Imager-A1, Zeiss, Gottingen, Germany). The spindle images were captured using Q capture software (Media Cybernetics, Inc., USA), and the spindle morphology was analyzed.

### Parthenogenetic oocyte activation

Parthenogenetic activation of in vitro matured oocytes was performed as previously described with slight modifications (Ma *et al.*, 2005). Oocytes were incubated with freshly prepared 10 mM strontium chloride (StCl_2,_ Cat No. 107865, Merck, India) in Ca^2+-^ and Mg^2+-^free M16 media supplemented with 0.1% BSA for 1 h. After incubation at 37°C in 5% CO_2_, the washed oocytes were cultured for 2 h in 30 µL of prewarmed M16 medium. After 2 h of culture, the oocytes were evaluated under an inverted phase contrast microscope (40X, IX 73, Olympus, Japan) for the presence of one pro nucleus (PN) and two extruded polar bodies in the perivitelline space, which confirmed oocyte activation.

### Statistical analysis

The data obtained in this study are from a minimum of 3 independent trials and are represented either as the mean±standard error of the mean (SEM) or as a percentage (%). Differences between the groups were tested either by one-way analysis of variance (ANOVA) or the Kruskal‒Wallis test depending on the distribution of the data. The chi-square test was applied when the data were in percentage form. Differences were considered significant when *p*<0.05. GraphPAD InStat software (GraphPad Inc., La Jolla, CA, USA) was used for statistical evaluation, and Microcal Origin 6.0 software (Origin Lab Corporation, Northampton, MA, USA) was used to prepare the graphs.

## RESULTS

### PRDL administration resulted in comparable oocyte maturation and improved the mitochondrial potential

**Figure 1:**
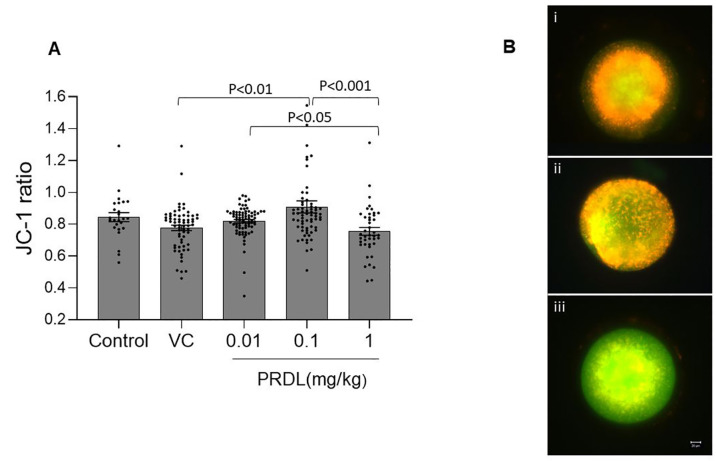
Mitochondrial membrane potential of oocytes after PRDL administration. A. Comparison of mitochondrial potential by JC-1 staining in control (n=25), VC (n=65), 0.01 (n=79), 0.1 (n=66) and 1mg/kg (n=42) PRDL-treated oocytes. B i-iii. Representative fluorescence microscopy images (40×) of JC-1-stained oocytes in decreasing order of mitochondrial potential. Scale bar =20 µm.

To mimic the clinical scenario, PRDL was administered for a total of three days, beginning on the day of PMSG administration and ending on the day of hCG injection. The retrieved COC was denuded and assessed for maturity. We observed a decrease in the average number of oocytes retrieved in the treated groups as the dose of PRDL increased. However, the difference was not statistically significant (Table 1). Although the 1 mg/kg PRDL group had the lowest number of oocytes (29.14±5.94), the highest maturation rate was also observed in the same group (83.57±7.87), which was comparable to that of the control group (77.11±9.79). These oocytes were further subjected to JC1 staining to determine the effect of PRDL administration on the mitochondrial potential of the oocytes. The JC1 ratio was significantly lower in oocytes from the 1 mg/kg PRDL group (0.75±0.02) than in those from the 0.01 mg/kg PRDL group (0.81±0.01; *p*<0.05; Fig. 1A) and 0.1 mg/kg PRDL group (0.91±0.01; *p*<0.001). The 0.1 mg/kg PRDL group even had a significantly greater JC1 ratio than the VC group (0.77±0.01; *p*<0.01). However, none of the PRDL-exposed groups showed significantly altered mitochondrial potential compared to that of the control group. Representative images of oocytes displaying various levels of mitochondrial potential are provided in Fig. 1B.

**Table 1:** Number of oocytes retrieved and maturation details from PRDL exposed groups.

Group	Number of oocytes retrieved (n)	Number of mature oocytes (n)	Maturation rate (%)
Control	30.28±10.12	23.28±9.31	77.11±9.79
Vehicle control (VC)	36.43±8.26	29.42±7.89	76.85±5.92
0.01mg/kg PRDL	49.29±14.11	31.85±8.66	69.5±5.04
0.1mg/kg PRDL	44.71±9.29	23.85±6.9	62.24±13.69
1mg/kg PRDL	29.14±5.94	24.42±5.68	83.57±7.87

Data are expressed as mean ± Standard Error of Mean from minimum of three trials conducted.

### PRDL administration did not alter oocyte developmental competence or apoptotic index in the blastocyst

**Figure 2:**
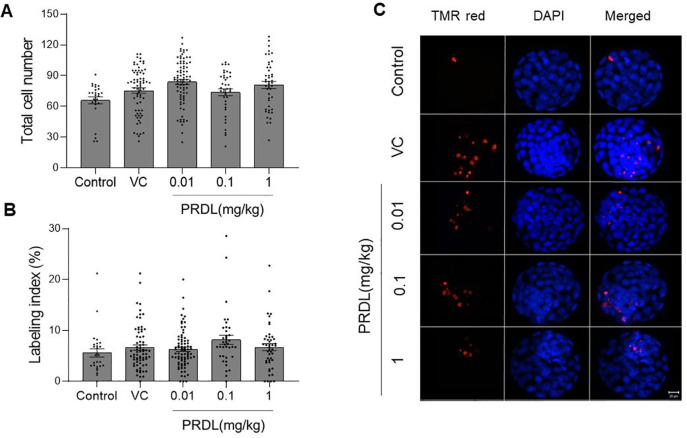
Impact of PRDL on blastocyst cell number and DNA integrity. A. Comparison of total cell numbers and B. Labeling indices of blastocysts from the control (n=27), VC (n=70), 0.01 (n=80), 0.1 (n=38) and 1 mg/kg (n=45) PRDL groups. C. Representative fluorescence microscopy (40×) images showing TUNEL-positive cells (red) and total cells (blue). Scale bar =20 µm.

To understand the developmental competence of PRDL-exposed oocytes, COCs isolated post superovulation were subjected to IVF. Complete details regarding fertilization and embryo development are presented in Table 2. However, after 10 hpi, the control group demonstrated the highest fertilization rate (91.07±4.98) among the study groups, but the difference was not statistically significant. Further progression of fertilized oocytes was monitored up to the blastocyst stage (96 hpi), and embryo progression was evaluated. Embryos derived from the PRDL group showed a comparable embryo development rate to that of control embryos. Even the blastocyst rate and hatching rate were comparable at 96 hpi. Although the rates of embryo progression in the PRDL group were similar to those in the control group, the total cell number (TCN) increased in blastocysts derived from oocytes in the 0.01 (*p*<0.001) and 1 mg/kg PRDL groups (*p*<0.05) compared to that in the control group (Fig. 2A). Furthermore, blastocysts obtained from each group were subjected to a TUNEL assay to assess DNA integrity. Interestingly, blastocysts across various study groups displayed comparable TUNEL indices, indicating that PRDL did not affect oocyte developmental competence or blastocyst quality (Fig. 2B). Representative images of blastocysts with TUNEL-positive cells are shown in Fig. 2C.

**Table 2:** Developmental competence of the oocytes exposed to prednisolone (PRDL) in vivo during superovulation protocol (number of oocytes/embryos in parentheses).

Group	Hours post insemination (hpi)								
	10	24	48		72		96		
	2PN/2PB	2 Cell	4 Cell & below	6-8 cell	Cleavage stage	Compacted	Early Blastocyst	Expanded	Hatching
Control (n=142)	91.07±4.98 (n=128)	96.87±3.13 (n=121)	50.26±12.52 (n=59)	49.73±12.52 (n=69)	5.9±2.9 (n=09)	94.1±2.96 (n=119)	16.9±2.13 (n=20)	22.97±10.23 (n=25)	46.87±10.99 (n=70)
Vehicle control(VC) (n=135)	75.7±13.68 (n=113)	78.87±9.12 (n=98)	52.46±6.89 (n=64)	47.53±6.89 (n=49)	23.76±8.5 (n=21)	76.23±8.50 (n=92)	5.96±3.23 (n=05)	15.6±3.37 (n=15)	51.27±9.09 (n=66)
0.01mg/kg PRDL (n=183)	61.27±11.02 (n=120)	93.73±5.25 (n=119)	49.03±0.52 (n=57)	51.61±1.13 (n=63)	4.93±2.94 (n=05)	96.06±3.4 (n=115)	39.33±8.24 (n=30)	22.3±7.04 (n=20)	36.06±16.65 (n=57)
0.1mg/kg PRDL (n=157)	74.93±7.75 (n=118)	90.17±6.38 (n=106)	34.4±14.38 (n=39)	65.6±14.38 (n=79)	14±2.81 (n=17)	86±2.80 (n=101)	15.83±3.56 (n=18)	28.8±5.46 (n=32)	40.7±5.55 (n=50)
1mg/kg PRDL (n=123)	83.76±8.19 (n=102)	97.33±1.41 (n=100)	28.86±11.23 (n=31)	71.13±11.23 (n=71)	3.76±1.94 (n=03)	96.23±1.94 (n=99)	8.5±3.21 (n=08)	17.03±6.4 (n=16)	70.23±10.99 (n=73)

Data are expressed as numbers and Mean ± Standard Error of Mean from minimum of three trials conducted.

### Direct exposure to PRDL did not affect the maturation or mitochondrial potential of oocytes matured in vitro but affected spindle morphology

**Figure 3:**
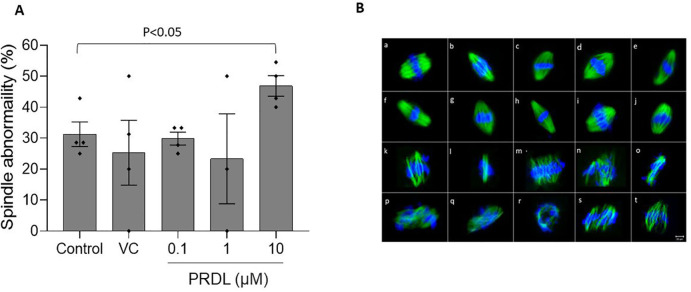
Spindle integrity of oocytes exposed to PRDL in vitro. A. Comparison of the percentage of spindle abnormalities in control (n=29), vehicle control (n=28), 0.1 µM (n=34), 1 µM (n=21), and 10 µM (n=36) PRDL-exposed MII oocytes. C. Representative fluorescence microscopy images (40×) of the spindle. (a-j) normal spindle arrangement; (k-t) damaged spindles and misaligned chromosomes. Scale bar =20 µm.

To assess the direct effect of PRDL, GV oocytes were subjected to IVM in the presence or absence of PRDL. PRDL at various concentrations (0.1, 1 and 10 µM) did not affect the oocyte maturation rate compared to that of control oocytes. The maturation rates between the groups were comparable. When these oocytes were subjected to JC1 staining to determine the effect of PRDL on oocyte mitochondria, the JCI ratio was comparable between the groups, indicating that PRDL did not affect the mitochondrial potential of the oocytes (Table 3). However, when the spindle morphology of these oocytes was assessed, the oocytes exposed to the highest PRDL concentration had a significantly greater incidence of abnormal spindle morphology than did the control oocytes (Fig. 3A). Representative images displaying normal and abnormal spindle morphology are shown in Fig. 3B. Furthermore, the activation rate of the oocytes exposed to PRDL was also comparable between the study groups.

**Table 3:** Effect of PRDL treatment on in vitro oocyte maturation and other tested parameters.

	Maturation rate (%)	JC1 ratio	Activation rate (%)
Control	52.96±2.44 (n=566)	0.68±0.06 (n=44)	89.05±10.95 (n=51)
Vehicle control (VC)	46.93±2.51 (n=598)	0.66±0.02 (n=42)	79.9±10.61 (n=45)
0.1 µM PRDL	48.04±2.62 (n=541)	0.58±0.03 (n=41)	87.97±2.92 (n=49)
1 µM PRDL	51.37±2.88 (n=571)	0.65±0.02 (n=48)	81.17±4.67 (n=41)
10 µM PRDL	46.76±3.80 (n=616)	0.60±0.06 (n=49)	68.12±22.94 (n=36)

Data are expressed as mean± standard error of mean from minimum of three trials.

## DISCUSSION

Although PRDL is used in routine clinical practice, there are limited experimental studies that have elucidated the effect of PRDL exposure on oocytes and embryos. This study demonstrated that short-term PRDL exposure to mouse oocytes at a therapeutic dose does not have a deleterious effect on oocyte competence when it is administered in vivo. However, direct exposure to high concentrations of PRDL can affect oocyte spindle morphology. This is the first experimental study to assess the impact of PRDL on mouse oocyte functional, genetic and developmental competence.

Initial reports on the administration of GCs alone or in combination with other adjuvants suggested that GCs improve the ovarian response during COS (Keay *et al.*, 1997; Trott *et al.*, 1996). It has been proposed that GC therapy might improve follicular development by suppressing androgen levels and increasing the production of growth factors, which are known to amplify the action of gonadotropins (Langford & Miell, 1993). Clinical studies involving PRDL administration during COS have demonstrated conflicting results with respect to oocyte yield and maturation potential (Revelli *et al.*, 2008; Ubaldi *et al.*, 2002). Notably, these studies included patients with good prognosis. In this study, a marginal reduction in oocyte yield was observed with an increasing PRDL dose. However, comparable maturation potential was observed between the PRDL-exposed oocytes and the control group. In mice, the administration of glucocorticoids such as cortisol and corticosterone did not affect oocyte maturation potential (Li *et al.*, 2018; Yuan *et al.*, 2016). However, a positive correlation was observed between the follicular fluid cortisol concentration and the morphological maturity of the follicles in women undergoing IVF (Fateh *et al.*, 1989).

Mitochondrial potential is an important indicator of oocyte quality because mitochondria are important organelles involved in various embryological processes, from fertilization to embryonic development (Tan *et al.*, 2017). Despite having comparable maturation rates, the 1mg/kg PRDL group showed significantly reduced mitochondrial potential compared to the other PRDL groups used in this study. This finding aligns with an earlier study in which the oocyte mitochondrial potential decreased with a simultaneous increase in oxidative stress when female mice were exposed to 50 mg/kg cortisol (Yuan *et al.*, 2020). However, a low concentration of PRDL had a positive effect on the oocyte mitochondrial potential. These differences could be due to variations in the chemical nature of the drug, the concentrations used (0.01, 0.1 and 1 mg/kg PRDL) and differences in study design.

To understand the developmental potential of oocytes treated with PRDL in vivo, we performed IVF. PRDL treatment (0.01-1 mg/kg) did not affect the embryo development rate. PRDL-treated oocytes had comparable blastocyst formation rates and hatching rates at 96 hpi. Similarly, the rate of blastocyst formation in oocytes treated with corticosterone did not differ from that in untreated oocytes (Li *et al.*, 2018). However, the developmental potential of oocytes decreased significantly when cortisol (50 mg/kg) was administered to female mice (Yuan *et al.*, 2016; 2020). Furthermore, earlier reports demonstrated a decreased blastocyst formation rate when oocytes/embryos were treated with high concentrations of GCs (Andersen, 2003; Čikoš *et al.*, 2019; González *et al.*, 2010a; 2010b; Uppangala *et al.*, 2021). These observed effects may be dose dependent and species specific, as it has been shown that exposing bovine oocytes to cortisol enhances their developmental competence (da Costa *et al.*, 2016). Furthermore, to assess blastocyst quality, the total cell number (TCN) was assessed. An increase in TCN in blastocysts was observed in the 0.01 and 1 mg/kg PRDL treatment groups, which is contrary to earlier reports in which comparable TCN was observed when cortisol (10 mg/kg) was injected into mice (Yuan *et al.*, 2016). Even in vitro studies have shown contradictory results for both comparable and decreased TCNs (Fateh *et al.*, 1989; Uppangala *et al.*, 2021).

GCs induce apoptosis as an immunomodulatory function (Herr *et al.*, 2007; Kogianni *et al.*, 2004; Schlossmacher *et al.*, 2011; Thouas *et al.*, 2004). GC decreases oocyte developmental potential by activating the Fas or TNFα system and inducing apoptosis in ovarian cells and oocytes (Yuan *et al.*, 2016; 2020). Previous studies have demonstrated that exposure of preimplantation embryos to various GCs, including PRDL, leads to increased apoptosis at high concentrations (Čikoš *et al.*, 2019; Uppangala *et al.*, 2021). This apoptotic process is considered a protective mechanism at the blastocyst stage, safeguarding embryos from potential DNA damage (Adiga *et al.*, 2007; Shimura *et al.*, 2002). Although a comparable TUNEL index was observed in blastocysts derived from PRDL-exposed oocytes in the present study, the detrimental effects of even low concentrations of GCs cannot be dismissed in vivo. The natural glucocorticoids present in our body may have synergistic effects on oocytes/embryos and may be present at high concentrations under various pathological conditions, such as oxidative stress conditions. Thus, inducing apoptosis and hampering embryo development indirectly by triggering Fas system-induced apoptosis in oviductal cells (Tan *et al.*, 2017).

Direct exposure of mouse oocytes to PRDL (0.1, 1, or 10 µM) during in vitro maturation resulted in comparable maturation rates. Similar observations were reported in earlier studies where dexamethasone and cortisol did not influence mice or bovine oocyte maturation or developmental competence (Andersen, 2003; Barroso *et al.*, 2020; da Costa *et al.*, 2016). Nevertheless, negative effects of cortisol and dexamethasone were observed in lamb and pig oocyte maturation (González *et al.*, 2010a; 2010b; Yang *et al.*, 1999). These observations indicate the species-specific effect of GCs; although mouse oocytes express GC receptors, GCs, including PRDL, do not influence mouse oocyte maturation. Even the mitochondrial potential of oocytes exposed to PRDL was like that of control oocytes. Similarly, earlier studies have shown that the mitochondrial distribution pattern and ultrastructure of mitochondria are unaffected when mouse and bovine oocytes are treated with corticosterone and dexamethasone (Barroso *et al.*, 2020; Li *et al.*, 2018).

Although direct exposure to PRDL did not affect the maturation rate or mitochondrial potential, oocytes treated with a high concentration of PRDL (at 10 µM) exhibited an increased incidence of spindle morphological abnormalities. Similarly, supplementation with a high concentration of dexamethasone (80 µg/mL) affected spindle morphology in mouse preantral follicle culture (Van Merris *et al.*, 2007). Normal meiotic spindle morphology in mature oocytes is crucial for maintaining their genetic integrity and development (Cimadomo *et al.*, 2021). Although this study did not observe an inverse relationship between increased abnormal spindle morphology and reduced maturation, as in the case of Van Merris *et al.* (2007), there was a decrease in the activation potential of oocytes exposed to 10 µM PRDL. However, the difference was not statistically significant.

ART procedures in which a low concentration of prednisolone is administered to improve IVF cycle outcomes may involve PRDL treatment for various periods (Andersen, 2003; Duvan *et al.*, 2006; Geva *et al.*, 2000; Revelli *et al.*, 2008; Robertson *et al.*, 2016), exposing the follicles/oocytes to PRDL for either the short or long term. This study attempted to replicate the clinical situation in which PRDL is treated during the short-term COS protocol. However, the differences observed between human and mouse models limit the extrapolation of the current findings to clinical settings. In addition, the implantation potential and gene expression of pluripotency markers in blastocysts derived from PRDL-exposed oocytes were not studied, and the developmental competence of oocytes subjected to PRDL treatment in vitro was not assessed, which are limitations of this study.

## CONCLUSION

In conclusion, short-term treatment with PRDL at a therapeutic dose does not have a deleterious effect on oocyte yield or developmental competence. However, direct treatment of oocytes with supraphysiological concentrations of PRDL can affect mouse oocyte spindle morphology. Further research is required to understand the effect of long-term exposure to PRDL on oocyte developmental competence and the clinical value of these observations on human oocyte developmental competence.
